# A novel *ex vivo* protocol that mimics length and excitation changes of human muscles during walking induces force losses in EDL but not in soleus of *mdx* mice

**DOI:** 10.1371/journal.pone.0320901

**Published:** 2025-04-07

**Authors:** Xiao Hu, Allison N. McCrady, Katherine E. Bukovec, Claire Yuan, Emily Y. Miller, Rachel K. Bour, Anthony C. Bruce, Katherine B. Crump, Shayn M. Peirce, Robert W. Grange, Silvia S. Blemker

**Affiliations:** 1 Department of Biomedical Engineering, University of Virginia, Charlottesville, Virginia, United States of America; 2 Department of Human Nutrition, Foods, and Exercise and Metabolism Core, Virginia Tech, Blacksburg, Virginia, United States of America; 3 Department of Orthopedic Surgery, University of Virginia, Charlottesville, Virginia, United States of America; 4 Department of Mechanical and Aerospace Engineering, University of Virginia, Charlottesville, Virginia, United States of America; University of Minnesota Medical School, UNITED STATES OF AMERICA

## Abstract

Although eccentric contraction protocols are widely used to study the pathophysiology and potential treatments for Duchenne muscular dystrophy (DMD), they do not reflect the stresses, strains, strain rates, and excitation profiles that DMD muscles experience during human daily functional tasks, like walking. This limitation of eccentric contractions may impede our understanding of disease progression in DMD and proper assessment of treatment efficacy. The goals of this study were to examine the extent of force loss induced by a gait cycling protocol we developed, and compare to that from a typical eccentric contraction protocol in soleus and extensor digitorum longus (EDL) muscles of *mdx* mice. To achieve this goal, *mdx* soleus and EDL muscles were subjected to eccentric contractions at three levels of strain (10%, 20% and 30% optimal length L_o_) and up to 200 cycles of our gait cycling protocol that mimicked the length changes and excitation patterns of the corresponding muscles during human walking gait. Our results showed that EDL but not soleus muscles had significant losses in isometric tetanic forces after the cycling protocols. Compared to the eccentric contraction protocol, the decrements in contractile performance from the cycling protocol were similar to those from the eccentric contractions at 10% in soleus and 20% L_o_ in EDL. Together, these results indicated the gait cycling protocol is a valuable experimental approach to better understand disease progression and to screen and evaluate efficacy of novel therapeutics for DMD.

## Introduction

Duchenne muscular dystrophy (DMD) is a severe monogenic disorder that leads to a nonfunctional dystrophin protein and subsequent loss of the dystrophin glycoprotein complex (DGC) from the sarcolemma of muscle cells [1,2]. The loss of the DGC results in progressive muscle degeneration and weakness, which eventually causes cardiorespiratory failure and death in patients with DMD. Although substantial advances in therapeutic treatments have been made especially in recent years, there are still challenges to overcome before effective treatments may be developed [1,3,4]. One of the challenges is poor translation of promising outcomes from preclinical studies in animal models of DMD to treatment for patients with DMD. The ineffective translation may be largely due to physiological and biomechanical differences between the animal models and humans [5,6].

Eccentric contraction protocols [7–10] and treadmill exercises [11–13] to characterize dystrophic muscle in mouse models of DMD, such as the *mdx* mouse, have been widely used to study both the pathophysiology and potential treatments for DMD. *Ex vivo* eccentric contraction protocols lengthen muscles at a given rate while the muscles contract in response to a set electrical stimulation frequency to impose acute stresses. Although eccentric contraction protocols have helped advance the understanding and treatments of DMD, they do not reflect the stresses, strains, strain rates, and activation profiles that dystrophic muscles of human patients experience during daily repeated functional tasks, like walking. For example, in most eccentric contraction protocols, although the ranges of lengthening (usually under 30% of optimal length L_o_) are generally within those encountered in walking [14], near maximal force production at a constant frequency of stimulation is typically applied, as muscles are lengthened at a fixed strain rate. In contrast, human muscles have time-varying force production and rate of length changes during walking, running, and moving up or down stairs; furthermore, force production only occasionally reaches a maximum [15,16]. During treadmill exercises, mice – as quadrupeds – employ more flexed postures during walking, which leads to substantially smaller strains in muscles compared to what human muscles may experience in walking [6]. These fundamental differences in force production and length change between *ex vivo* eccentric contraction protocols, treadmill exercises and human *in vivo* muscle function during daily tasks may impede our understanding of disease progression in DMD and limit proper assessment of treatment efficacy by relying primarily on *ex vivo* eccentric contraction protocols. Thus, it will be beneficial to replicate *in vivo* muscle activation (excitation), stresses and strains *ex vivo*.

Recently, we developed a novel *ex vivo* protocol to mimic muscle function in human daily tasks in dystrophic muscles in mouse models of DMD [17]. This protocol scales and adjusts the time-varying lengths and excitations experienced by muscles during human walking, so it can be mimicked in smaller isolated mouse muscles with equipment similar to that used for *ex vivo* eccentric contraction protocols. The time-varying muscle lengths and excitations are obtained from computer simulations of movement with detailed musculoskeletal models developed based on extensive imaging and dissection measurements [18,19]. In our proof-of-principle study [17], wild-type (WT) and *mdx* mice soleus muscles closely mimicked *in vivo* soleus muscle function during walking and well-tolerated 25 cycles of human walking gait, both of which indicated the feasibility of the protocol. However, it remains unclear how dystrophic muscles may respond to a larger number of repeated gait cycles *ex vivo*, as experienced during human daily walking, and how the responses of dystrophic muscles to this new protocol may differ from those of typical *ex vivo* eccentric contraction protocols.

The goal of this study was to test two hypotheses about the *ex vivo* cycling protocol. First, because the function of human lower limb muscles during walking may contribute to muscle degeneration in patients with DMD [6,20], we hypothesized that dystrophic soleus and extensor digitorum longus (EDL) muscles from *mdx* mice will demonstrate force loss when exposed to an *ex vivo* gait cycling protocol of several hundred gait cycles. Second, because our proof of concept study demonstrated limited damage in the *mdx* soleus subjected to 25 gait cycles [17], we hypothesized that the damage to *mdx* soleus and EDL muscles that resulted from the *ex vivo* gait cycling protocol would be less severe than a typical *ex vivo* eccentric contraction protocol [9] for either soleus or EDL muscles. To assess these two hypotheses, up to 200 walking cycles were mimicked in soleus and EDL muscles using our *ex vivo* protocol of gait cycling [17] that scales the length changes of muscles based on architectural measurements and uses voltage modulation to excite mouse muscles in patterns close to human muscles in walking. Then, the muscle responses to this protocol were compared to those from an eccentric contraction protocol [9]. Our results highlight the differences between mouse and human muscle mechanics and the potential of the *ex vivo* gait cycling protocol as a valuable experimental approach to evaluate treatment efficacy and develop novel therapeutics for patients with DMD.

## Methods

### Animal and muscle preparation

All mouse protocols were approved by the Institutional Animal Care and Use Committee at Virginia Tech. Mice were fed standard chow, provided water ad libitum, and maintained on a 12-h light-dark cycle. Soleus and extensor digitorum longus (EDL) muscles from male C57BL/10ScSn-Dmd*mdx*/J mice at age 5–10 (7.7 ±  1.0) weeks (*mdx*; stock no. 001801, Jackson Laboratories), and C57BL/10ScSnJ mice at age 6–10 (8.0 ±  0.8) weeks (WT; stock No. 000476, Jackson Laboratories), were subjected to either an *ex vivo* eccentric contraction protocol [9] or an *ex vivo* gait cycling protocol that mimicked human walking gait [17]. The ages of the mice were chosen in our experiments because the literature showed that the degeneration and regeneration of hindlimb muscles of *mdx* mice are largely stabilized after ~ 6 weeks [21–23].

Mice were deeply anesthetized by an intraperitoneal injection of ketamine-xylazine (2 mg of ketamine-20 mg xylazine/100 g body mass), and once a deep plane of anesthesia was achieved, dissection began. The right and left soleus or EDL muscles were carefully dissected and 4-O suture tied to both proximal and distal myotendinous junctions [24]. The muscles were immersed in a jacketed water bath of physiological saline solution (PSS) maintained at 30°C [24]. Muscles from one side would undergo either eccentric contraction or gait cycling protocols (see the sections below), while those from the other side would serve as unstimulated controls that stayed in the experimental apparatus for the same duration. The unstimulated muscles provided information to check whether there were apparent damages to the muscle during the process of dissection and protocols. Mice were euthanized with an intracardiac injection of ketamine-xylazine, followed by cervical dislocation.

### Pre-protocol contractions and measurements

Before the start of either the eccentric or the gait cycling protocols, the dissected soleus and EDL muscles were hung by clamping the distal suture at the bottom of the bath and the proximal suture tied to the arm of the dual-mode servomotor system (Aurora 300C-LR). Optimal muscle length (i.e., L_o_) for soleus and EDL were determined as described [24]. Briefly, muscle resting tension was adjusted as an index of muscle length until the maximal twitch response was elicited [24]. A resting tension of 10 mN for both muscles yielded the maximum twitch response. All muscles were set to a resting tension of 10 mN followed by pre-contractions. Both the soleus and EDL muscles were stimulated to contract via platinum wire electrodes ~ 2mm either side of the muscle. After 10 min of quiescence, 3 twitches (1 min apart) and 3 tetani at 150 Hz (1 min apart) were elicited at 30V. Resting tension was reset to 10 mN after each contraction. The muscle resting tension was stable for subsequent assays. Calipers were used to measure muscle length to the nearest 0.1mm. After another 10 min of quiescence, the pre-force frequency was conducted at L_o_, with frequencies of stimulation varied from 1 Hz (twitch) to 180 Hz, with 1 min between each stimulation. In the pre-force frequency, the maximal isometric force was recorded.

### Eccentric contraction protocol

**Fig 1:**
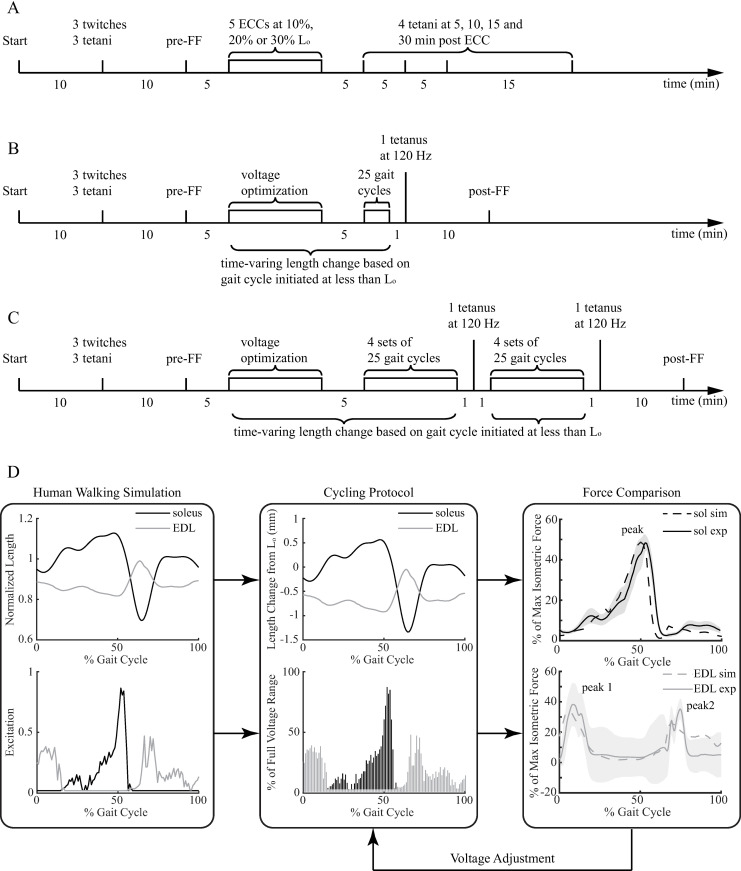
The timelines of the eccentric contraction **(A), 25-cycle (B) and 200-cycle (C) gait cycling protocols.** The protocol steps that were initiated at less than L_o_ were indicated in (B and C). The overview of the gait cycling protocol is shown in (D). sol: soleus; sim: simulation; exp: experiment.

An eccentric contraction protocol was followed as described [8,9] with some changes as noted below (Fig 1A; S1 Fig). Both WT and *mdx* soleus and EDL muscles were subjected to 5 eccentric contractions, with 4 min quiescence at L_o_ between each. For each eccentric contraction, both soleus and EDL muscles were stimulated at 80 Hz for a 500 ms isometric contraction, followed by a lengthening at 0.5 L_o_/s for another 200 ms, 400 ms or 600 ms to yield a specific displacement of 10%, 20% or 30% L_o_, respectively. Mice were divided into 3 different groups and muscles within each group were stretched to either 110%, 120% or 130% L_o_ to yield total strains of 10%, 20% or 30% L_o_ in eccentric contractions, respectively. At 5, 10, 15 and 30 min after the final stretch, tetanic (80 Hz) responses from the muscle were elicited to assess force recovery. After the final tetanic contraction, the muscles were incubated in a 0.2% procion orange/PSS solution (Reactive Orange 13, product number S477583-50MG, Sigma-Aldrich) for 60 min to assess membrane leakiness. Muscles were then washed two times, each time for 5 min in fresh PSS and lightly blotted; tendons were dissected free, and the mass to the nearest 0.1 mg was determined on an A-200D electronic analytical balance (Denver Instruments). Muscles were mounted for sectioning as described below.

### Gait cycling protocol to mimic muscle function during human walking gait

The gait cycling protocol used to mimic muscle function during human walking gait was based on a dynamic musculoskeletal simulation of walking of a healthy human subject developed by John et al. [25] (https://simtk.org/projects/muscleprops) The technical details of this protocol were described in [17]. A brief description of the protocol is summarized below (Fig 1D).

The dynamic walking simulations reflected a healthy male (height: 1.83 m; weight: 65.9 kg) walking at 1.36 m/s on an instrumented treadmill (cadence: 109 steps/min; stride length: 1.45 m). A three-dimensional musculoskeletal model of human lower limbs [26,27] consisting of 12 body segments, 19 degrees of freedom, and 92 muscle-tendon units was used to develop this simulation in OpenSim [28]. Each muscle tendon unit was modeled as a Hill-type muscle model with muscle-specific architectural and geometric parameters to characterize its contraction dynamics and force-generating capacity [29]. From this simulation, time-varying values of excitation, fiber length, and force of soleus and EDL in one gait cycle were extracted to serve as inputs to the gait cycling protocol.

To mimic function of human muscles in mouse muscles with much smaller sizes, scaling based on muscle architectural parameters was applied. Specifically, the normalized lengths of muscles (either soleus or EDL) at each time instant *t* was calculated by:


$$\tilde{L}\left(t\right)=\frac{L^{human}\left(t\right)}{L_{o}^{human}}$$ (1)

where $L^{human}\left(t\right)$ is the simulation-predicted length of a human muscle fiber at time instant *t* during one gait cycle, and $L_{o}^{human}$ (obtained from the lower limb musculoskeletal model) is the optimal fiber length of either soleus or EDL muscle. Then, the length changes with respect to the optimal fiber length in mouse muscle $\Delta L^{mouse}\left(t\right)$ were calculated as:


$$\Delta L^{mouse}\left(t\right)=\tilde{L}\left(t\right)\text{*}L_{o}^{mouse}-L_{o}^{mouse}$$ (2)

Where $L_{o}^{mouse}$ is the optimal fiber lengths of mouse soleus or EDL muscles [30]. The $\Delta L^{mouse}\left(t\right)$ would be used to vary mouse muscle length in the gait cycling protocol, which yielded length changes of about 40% and 20% L_o_ in soleus and EDL, respectively. Similarly, forces produced by human soleus or EDL muscles in walking gait were normalized by their corresponding maximum isometric forces in the lower limb model [26,27], and those by mouse soleus or EDL muscles were normalized by the corresponding measured maximum isometric forces in force-frequency stimulations. These normalized forces in human and mouse muscles were compared to adjust electrical stimulation intensity so that comparable forces were generated in mouse muscles in the gait cycling protocol.

To start the gait cycling protocol, muscles were first set and maintained at 10 mN resting tension (L_o_), while they equilibrated in the bath. After equilibration, the following steps were performed: 1) pre-twitches and tetani (150 Hz), 2) pre-force frequency, 3) voltage optimization for gait cycling, 4) gait mimicking, and 5) post-force frequency (Fig 1B and C). Steps 1 and 2 were identical to those in the eccentric contraction protocol. In Step 3, to determine the voltage needed to produce the appropriate levels of force in mouse soleus and EDL muscles when mimicking walking gait, the initial voltage range was set to 0–20 V with a scale factor of 1. If the desired force levels were not achieved within this range, the voltage range was set to 0–80 V, and a scaling factor was used to adjust the force levels until the desired levels were reached (see *Additional excitation voltage modulation* in [17]). Once Step 3 was completed, and after 5 min of rest, the soleus or EDL muscle underwent either 25 or 200 consecutive cycles mimicking walking gait at a rate of 1 cycle/s. The 200 cycles were completed in multiple sets of 25 cycles with a 1-min rest in between. During the protocol, the rate of stimulation was synchronized with the rate of muscle length change at 222 Hz to achieve a smooth trajectory of length change. One minute following the completion of the 25^th^ or 200^th^ gait cycle, a 120-Hz tetanus was delivered at L_o_. During the 200-cycle protocol an additional 120-Hz tetanus was elicited after the completion of the 100^th^ cycle to check the muscle viability at the middle of the 200 cycles. Muscles then rested for 10 min at L_o_, and the post-force frequency protocol was performed. The duration of the cycling protocols was within 1.5 hours. Note, all isometric and eccentric contractions were initiated with muscles at L_o_ (i.e., 10 mN) and all gait cycles were initiated with muscles at less than L_o_ to mimic the initial length of the human muscles (see Fig 1B and C) in a gait cycle.

### Histological analyses

Soleus and EDL muscles were incubated in procion orange dye prior to sectioning to identify leaky fibers [8,9]. After completion of the 0.2% procion orange incubation and subsequent washes, muscles were embedded in 30% sucrose and optimal cutting temperature compound and flash frozen. The tissue blocks were then sectioned in a cryostat (NX50 Cryostar). To define the sarcolemmal membranes, the 10- um thick cryosections were fixed in 4% PFA, permeabilized with 0.3% triton, blocked with 10% donkey serum, incubated with α-2-laminin primary antibody (Sigma-Aldrich #L0663, clone 4H8-2) for one hour at 22°C, and incubated with donkey anti-rat secondary antibody conjugated to Alexa Fluor 488 fluorophore (Thermo Fisher Scientific, catalog # A-21208) for one hour at 22°C. Images were captured at 10X using a Nikon Eclipse C1 point scanning confocal microscope (Nikon Metrology Inc., Brighton, MI), independently fluorescing the red (procion orange) and then the green dyes (laminin).

Image tiles for each stain (procion orange and laminin) were manually stitched together for the individual images. The laminin image was then imported into FIJI [31] and the color threshold was set to show the most complete fiber borders. The image was then binarized and any artifact fluorescence outside of the section was removed. The image was then transformed back to the green channel and exported to be overlaid with the procion orange image.

An automated image processing algorithm was developed in MATLAB (R2022b, MathWorks, Natick, MA) using user-defined segmentation parameters (i.e., adaptive thresholding sensitivity, number of outer layers removed, and gap removal to exclude any extraneous fibers segmented without boundaries present) to allow for visual optimization of the segmentations. The algorithm used contrast-limited adaptive histogram equalization to improve the contrast in the image [32]. The fiber image was then binarized using an adaptive threshold determined by the local mean intensity in the neighborhood surrounding the pixel [33]. This step was followed by enhancement methods to clear the image borders, and close pixel gaps in the fiber borders (green channel). The procion orange (red channel) was then binarized according to a global threshold [34] calibrated to the detectable amount of dye counted in 16 manual image segmentations by two trained analysts. The analysts were blind to the genotype and protocol group during segmentation. The fiber boundaries were then segmented using the binarized mask with 10 iterations of an active contouring algorithm [35] followed by a watershed transform [36] and then 15 iterations of the active contouring algorithm again. Watershed transform can lead to errors in segmentation due to noise within an image, therefore prior to transformation, the image was smoothed to suppress local minima. Multiple iterations of active contouring ensured smoothness of the fiber boundaries. Each fiber was then individually used as a mask on the binarized procion orange image, and the fiber was marked as positive for presence of dye if the area of procion orange fluorescence was greater than 50% of the fiber area. Following this analysis, the outer two layers of segmented fibers were removed due to the risk of damage during dissection, which may have resulted in high amounts of procion orange stain in those fibers. The percent of procion orange positive fibers (# positive fibers/# total fibers * 100%) were exported with the final segmented image for further analysis. The difference in percent positive fibers from the automated segmentations versus the manual segmentations were: Analyst 1 average difference =  2.4%, median difference =  0.7%, maximum difference =  18.6% and Analyst 2: average difference =  1.1%, median difference =  0.8%, maximum difference =  5.6%. These values are similar to the differences between manual segmentation percent positive fibers between the two analysts (average difference =  3.1%, median difference =  1.4%, maximum difference =  16.9%).

### Data processing and statistics

To compare the force losses and recoveries during the eccentric contraction protocols, the forces from each of five eccentric contractions and during recovery for each muscle were normalized to the forces from the first eccentric contraction for that muscle. When comparing force losses and recoveries during the gait cycling protocols, the dynamic peak forces from each gait cycle (Fig 1D) and the isometric tetani during recovery for each muscle were recorded and normalized to maximum isometric forces from that muscle’s pre-force frequency profile. Means and standard errors of the mean were then determined for either the groups of muscles subjected to the eccentric protocol or to the gait cycling protocols. The comparisons between the eccentric contraction protocols and the gait cycling protocols were made using the force responses measured at 80 Hz 10 minutes after the eccentric contractions were complete, and from the 80 Hz response obtained during the post-force-frequency 10 minutes after the gait cycling protocol was complete. The force responses 10 minutes after the completion of both protocols were selected for the comparison to minimize the potential effect of fatigue. All force responses at 80 Hz were normalized to the forces at 80Hz from the pre-force-frequency profile for either of the two protocols, which indicates the level of force losses after the protocols (i.e., normalized force smaller than 1 indicates a force loss) as a measure of damage to the contractile function of muscle [9,10,37]. All muscles for these comparisons had force responses elicited at L_o_.

Linear mixed-effects (LME) models [38] with genotypes and number of eccentric contractions (for the eccentric contraction protocol) or number of cycles (for the gait cycling protocol) as fixed effects and mice as a random effect were used to examine the effects of genotypes and eccentric contractions or gait cycles on the force losses in the respective protocols. Similar LME models with genotypes and time of the force measurements as fixed effects and mice as a random effect were used to examine the effects of genotypes and force recovery after the eccentric and gait cycling protocols. When comparing the cycling protocols and eccentric contraction protocols, LME models were used to examine the effect of protocols (as a fixed effect) on the recovery forces after the two protocols. LME models were used to account for the repeated measures (e.g., multiple eccentric contractions or gait cycles) from the same sample during protocols. The sequential Bonferroni correction (Holm-Bonferroni method) was used to control all the pairwise comparisons from each of the LME models [39]. These statistical analyses were conducted in Matlab (R2022b, Mathworks, Natick, MA, United States) with the significance level set at *p* <  0.05.

The percent of dye-positive fibers of each sample were used for final comparative analysis. Statistical outliers for each group were removed using the Dixon method [40]. For each variable, a two-way ANOVA (genotype, injury protocol) with a Tukey’s post hoc test was used. R Statistical Programming Software (v4.2.2, R Core Team 2021) and a significance level at *p* <  0.05 were employed.

## Results

### Eccentric contraction protocol

**Fig 2:**
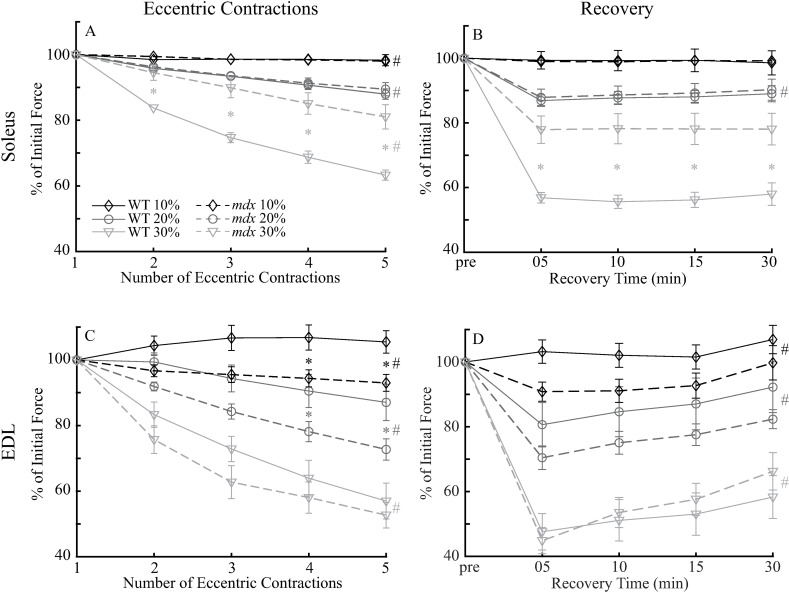
The force reduction resulted from a series of five eccentric contractions at 10%, 20% and 30% L_o_ from WT and*mdx* soleus (A) and EDL (C) and recovery of isometric tetanic force following eccentric contraction protocol in WT and *mdx* soleus (B) and EDL (D) muscles. All forces were normalized to the isometric force before the 1st eccentric contraction. Data shown are mean ±  SEM. The SEMs smaller than the symbols are not shown. *  differences between WT and *mdx*. # significant main effects of number of eccentric contractions (A and C) and recovery time (B and D) in both WT and *mdx* mice. All comparisons *p* <  0.05. pre: the isometric force before the 1st eccentric contraction. Soleus: 10% L_o_, n =  10 for WT and *mdx*; 20% L_o_, n =  10 for WT and *mdx*; 30% L_o_, n =  8 for WT and 9 for *mdx*. EDL: 10% L_o_, n =  10 for WT and 8 for *mdx*; 20% L_o_, n =  10 for WT and *mdx*; 30% L_o_, n =  15 for WT and 14 for *mdx*.

As previously reported in the literature, *mdx* soleus muscle tolerated eccentric contractions similar to WT soleus muscle, while *mdx* EDL muscle generally had greater force losses due to the eccentric contractions compared to WT EDL muscle. After five eccentric contractions at 10% and 20% L_o_, soleus muscles in both genotypes only lost about 2% and 10% of the initial force, respectively (Fig 2 A). There were no differences in force responses between the WT and *mdx* soleus muscles (all *p* >  0.3) at either 10% or 20% L_o_. The eccentric contractions at 30% of L_o_ led to 38% and 19% losses of force in WT and *mdx* soleus, respectively, with *mdx* soleus showing significantly less force loss from contractions 2–5 (all *p* <  0.01). In contrast to soleus, *mdx* EDL muscle showed significantly greater force losses from eccentric contractions 3–5 at 10% L_o_ (all *p* <  0.05) and from eccentric contractions 4 and 5 at 20% L_o_ (both *p* <  0.05; Fig 2C). During eccentric contractions at 30% L_o_, EDL muscles from both genotypes lost comparable amounts of force (all *p* >  0.3) that reached over 40% after five eccentric contractions.

After the eccentric contractions were complete, the soleus muscle generally did not show much recovery of force loss, whereas the EDL muscle showed significant recovery within 30 minutes. After eccentric contractions at 10% and 30% L_o_, the WT and *mdx* soleus muscles did not show recovery of isometric tetanic force following ECC within 30 minutes of the final eccentric contraction (e.g., from 05–30 min, both *p* >  0.7; Fig 2B). After eccentric contraction at 20% L_o_, although the recovery was statistically significant (*p* <  0.01), the actual recovery was only about 2%. There was no genotype difference in recovery of soleus at 10% and 20% L_o_ (both *p* >  0.7). However, after eccentric contractions at 30% L_o_, tetanic forces (at 80 Hz) of WT soleus were lower than those of *mdx* soleus at all four recovery time points (all *p* <  0.001). In contrast to the soleus muscle, the WT and *mdx* EDL muscles showed significant recovery at all three stretch magnitudes of eccentric contractions (all *p* <  0.01; Fig 2D). In addition, there was no difference in recovery between the two genotypes (all *p* >  0.05).

### Gait cycling protocol

**Fig 3:**
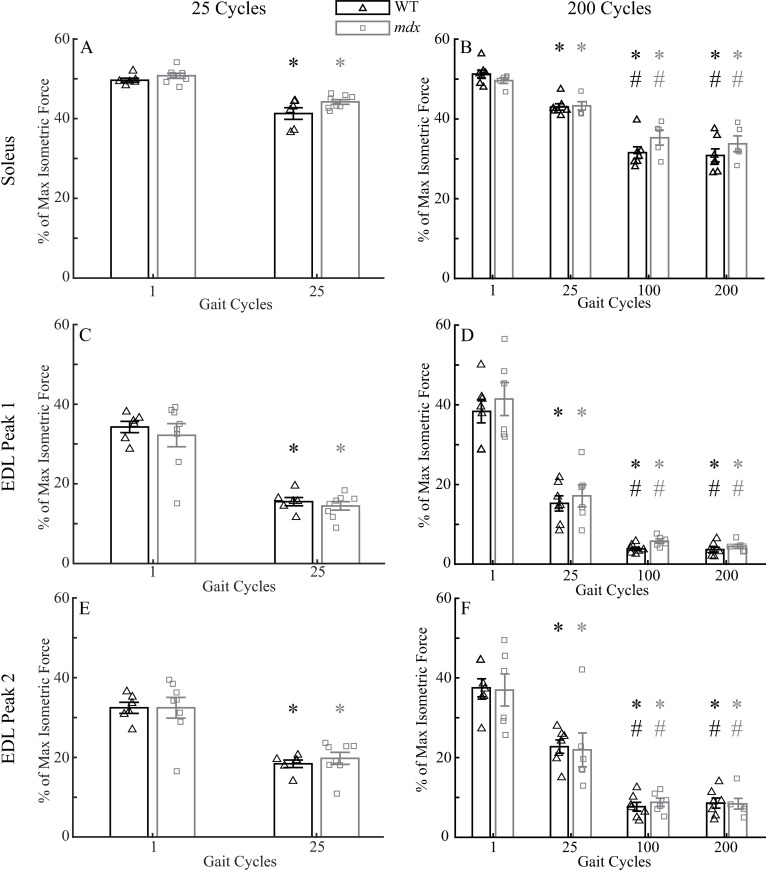
Comparisons of forces in soleus muscles in the 25-cycle **(A) and 200-cycle (B) protocols, and those in EDL muscles in the 25-cycle (C and E for Peak 1 and 2, respectively) and 200-cycle (D and F for Peak 1 and 2, respectively) protocols.** All forces were normalized to the maximum isometric tetanic force measured during the pre-force frequencies. Data shown are mean ±  SEM. *  differences from 1^st^ cycle at *p* <  0.01; # differences from 25^th^ cycle at *p* <  0.01. Soleus: 25-cycle, n =  6 for WT and 8 for *mdx*; 200-cycle, n =  7 for WT and 5 for *mdx*. EDL: 25-cycle, n =  6 for WT and 8 for *mdx*; 200-cycle, n =  7 for WT and 6 for *mdx*.

Soleus and the EDL muscles had significant force losses after undergoing gait cycling protocols that mimicked the length changes and activation profiles of these two muscles in human walking. In the 25-cycle protocol, peak forces of both the soleus and EDL muscles were reduced significantly at the 25^th^ cycle compared to the 1^st^ cycle (Fig 3A, 3C and 3E; soleus: *p* <  0.001; EDL: *p* <  0.001 for peak 1 and peak 2). There was no difference in force loss (difference in peak force between the 1^st^ and 25^th^ cycles) between the two genotypes for soleus (*p* =  0.21) or for EDL (peak1: *p* =  0.72; peak 2: *p* =  0.55). Similarly, in the 200-cycle protocol, peak forces of both the WT and *mdx* soleus and EDL muscles were reduced due to the repeated gait cycles (Fig 3B, 3D and 3F; soleus: *p* <  0.001; EDL *p* <  0.001 for peak 1 and peak 2). There was no difference in force loss between the two genotypes for soleus (25^th^ cycle: *p* =  0.31; 100^th^ cycle: *p* =  0.10; 200^th^ cycle: *p* =  0.18) or for EDL (25^th^ cycle: *p* =  0.73 for peak 1 and *p* =  0.93 for peak 2; 100^th^ cycle: *p* =  0.81 for peak 1 and *p* =  0.71 for peak 2; 200^th^ cycle: *p* =  0.64 for peak 1 and *p* =  0.94 for peak 2). There was a greater force loss at the 200^th^ cycle than at the 25^th^ cycle (soleus: WT and *p* <  0.01; EDL: both WT and *mdx p* <  0.01 for peak 1 and peak 2). The force loss largely plateaued after the 100^th^ cycle in both muscles. There was no difference in peak forces between the 100^th^ and 200^th^ cycles (soleus: WT *p* =  0.64, *mdx p* =  0.38; EDL: WT *p* =  0.94, *mdx p* =  0.62 for peak 1 and WT *p* =  0.73, *mdx p* =  0.90 for peak 2).

**Fig 4:**
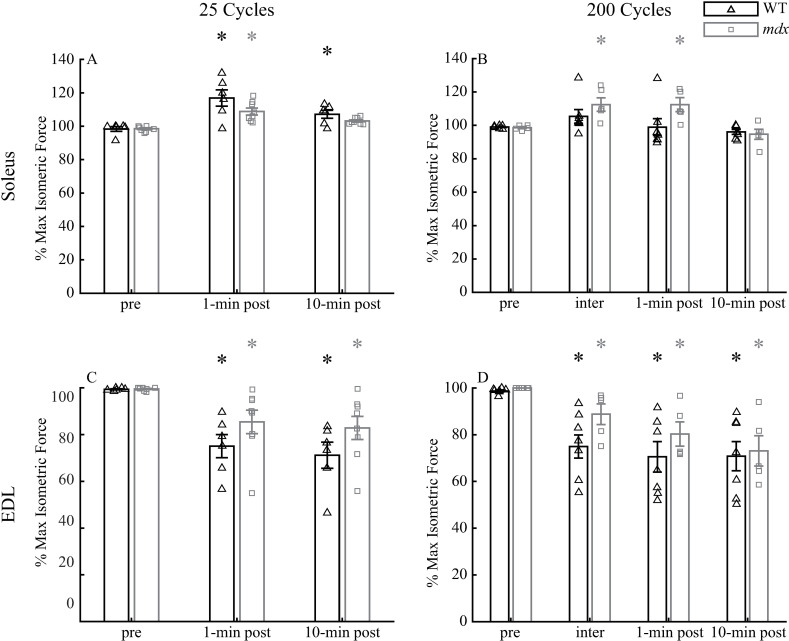
Comparisons of isometric tetanic forces at 120 Hz in soleus muscles in the 25-cycle **(A) and 200-cycle (B) protocols, and those in EDL muscles in the 25-cycle (C) and 200-cycle (D) protocols.** All forces were normalized to the maximum isometric tetanic force measured during the pre-force frequencies. Data shown are mean ±  SEM. * differences from pre at *p* <  0.05. pre: 120Hz tetanus during force-frequency before the protocol; 1-min post: a 120 Hz tetanus 1 minute after the protocol; 10-min post: a 120Hz tetanus during force-frequency 10 minutes after the protocol; inter: a 120 Hz tetanus after the 100^th^ cycle in 200-cycle protocol. Soleus: 25-cycle, n =  6 for WT and 8 for *mdx*; 200-cycle, n =  7 for WT and 5 for *mdx*. EDL: 25-cycle, n =  6 for WT and 8 for *mdx*; 200-cycle, n =  7 for WT and 5 for *mdx*.

Although the peak forces of both soleus and EDL were reduced during the gait cycling protocol, only the isometric tetanic forces (at 120Hz) of EDL were reduced after compared to before the gait cycling protocol, and this reduction of tetanic force did not recover 10 minutes after the protocol. Soleus muscles had increased maximum isometric forces 1 minute after the 25-cycle protocol compared to that before the protocol (both genotypes *p* <  0.001; Fig 4A). This enhanced isometric force decreased 10 minutes after the protocol in WT soleus (*p* <  0.01), but not in *mdx* (*p* =  0.09). However, WT soleus still demonstrated increased isometric force 10 minutes after the protocol than before the protocol (*p* <  0.01), while the isometric force of *mdx* soleus 10 minutes after was comparable to that before the protocol (*p* =  0.09). In contrast, EDL muscle had reduced maximum isometric forces 1 minute (both genotypes *p* <  0.01) and 10 minutes (both genotypes *p* <  0.01) after the 25-cycle protocol compared to those before the protocol (Fig 4C). Resting for 10 minutes did not significantly alter the levels of the maximum isometric forces in the EDL muscle (both genotypes *p* >  0.8). There were no genotype differences in soleus and EDL at all tested times (*p* >  0.1 for soleus and EDL) with the only exception in soleus at 1 minute after the protocol (*p* <  0.05).

The maximum isometric forces at 120 Hz in soleus and EDL muscles after the 200-cycle protocol followed similar patterns. One minute after the 200-cycle protocol, *mdx* soleus had an increased isometric force compared to that before the protocol (*p* <  0.05; Fig 4B). This increased force generating ability also existed in the middle (e.g., inter, at 120Hz) of the protocol (*p* <  0.05), but diminished 10 minutes after the protocol (*p* =  1). The WT soleus did not show an increased isometric force generating ability in the middle (e.g., inter, *p* =  0.49), 1 minute after (*p* =  0.99), and 10 minutes after (*p* =  1) the 200-cycle protocol. The EDL muscle had reduced maximum isometric forces in the middle of (e.g., inter, WT *p* <  0.001; *mdx p* =  0.15), 1 minute after (both genotypes *p* <  0.001), and 10 minutes after (both genotypes *p* <  0.001) the 200-cycle protocol compared to that before the protocol (Fig 4D). Resting 10 minutes did not have any effects on the isometric forces of EDL muscle (WT *p* =  0.95; *mdx p* =  0.48). There were no genotype differences for soleus or EDL at all tested times (*p* >  0.1 for soleus and EDL) with the only exception being soleus at 1 minute after the protocol (*p* <  0.05).

### Dye uptake

**Fig 5:**
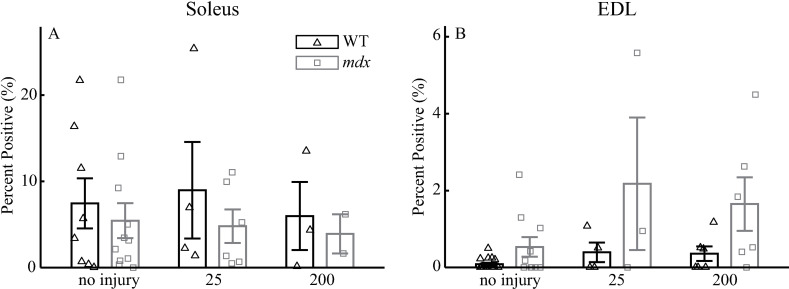
Comparisons of dye uptake after 25-cycle and 200-cycle protocols for Soleus **(A) and EDL (B)**. Data shown are mean ±  SEM. EDL muscles showed significant main effects among groups (no injury vs. 25 cycles vs. 200 cycles; *p* =  0.04) and between genotypes (WT vs. mdx; *p* <  0.01) after the cycling protocols. Soleus: no injury, n =  8 for WT and 11 for *mdx*; 25-cycle, n =  4 for WT and 6 for *mdx*; 200-cycle, n =  3 for WT and 2 for *mdx*. EDL: no injury, n =  16 for WT and 10 for *mdx*; 25-cycle, n =  4 for WT and 3 for *mdx*; 200-cycle, n =  6 for WT and 6 for *mdx*.

The gait cycling protocols did not alter the amount of dye uptake in WT and *mdx* soleus and EDL muscles. After the cycling protocols, the soleus muscles showed no differences among the groups (no injury vs. 25 cycles vs. 200 cycles; *p* =  0.94) and between genotypes (WT vs. *mdx*; *p* =  0.31; Fig 5A). Although the EDL muscles showed significant main effects among groups (no injury vs. 25 cycles vs. 200 cycles; *p* =  0.04) and between genotypes (WT vs. mdx; *p* <  0.01) after the cycling protocols (Fig 5B), the 25-cycle protocol did not increase the dye uptake in WT (*p* =  0.99) and *mdx* EDL (*p* =  0.17) muscles compared to the no-injury group. Similarly, the 200-cycle protocol did not increase the dye uptake in WT (*p* =  0.99) and *mdx* EDL (*p* =  0.30) muscles compared to the no-injury group.

### Comparison between eccentric and cycling protocols

**Fig 6:**
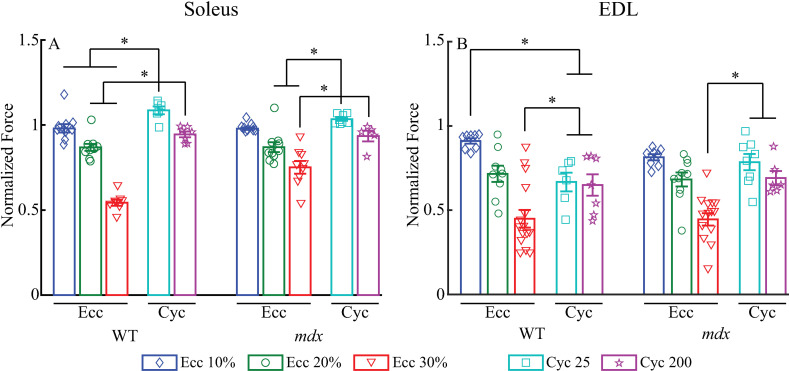
Comparisons between the cycling and eccentric contraction protocols in soleus **(A) and EDL (B) muscles**. All forces were from 80Hz tetani 10-minute after the protocols and normalized to forces of the 80 Hz tetani before the corresponding protocols. Data shown are mean ±  SEM. *  differences between compared protocols (*p* <  0.05).

When comparing the tetanic forces (at 80Hz) 10-minutes after the conclusion of the protocols normalized to values before the protocols, soleus and EDL muscles that underwent the gait cycling protocols largely showed higher or comparable forces compared to those that underwent the eccentric protocols. After the 25-cycle protocol, the WT and *mdx* soleus muscles showed higher normalized forces than those after eccentric contraction protocols (WT: *p* <  0.01 for 25 cycles vs. eccentric contractions at 10%, 20%, and 30% L_o_; *mdx*: *p* <  0.01 for 25 cycles vs. eccentric contractions at 20% and 30% L_o_; Fig 6A). The only exception was the *mdx* soleus after the eccentric contraction at 10% L_o_, which had comparable forces to those after 25-cycle protocol (*p* =  0.36). For EDL muscles, normalized forces 10 minutes after the 25-cycle were comparable to those after eccentric contraction protocol at 10% L_o_ for *mdx* (*p* >  0.05) and at 20% L_o_ for both WT (*p* >  0.3) and *mdx* (*p* >  0.3; Fig 6B). Normalized forces were higher than those at 30% L_o_ (*p* <  0.05 for WT and *mdx*). Only normalized forces of WT EDL after the 25-cycle were lower than after the eccentric contraction at 10% (*p* <  0.05). After the 200-cycle protocol, the WT soleus muscles had higher forces than those after eccentric contraction at 20% L_o_ (*p* <  0.05) and 30% L_o_ (*p* <  0.01), but comparable forces to those after eccentric contraction at 10% L_o_ (*p* >  0.3; Fig 6A). Similarly, the *mdx* soleus muscles had higher forces than those after eccentric contraction at 30% L_o_ (*p* <  0.01), but comparable forces to those after eccentric contraction at 10% L_o_ (*p* >  0.3) and 20% L_o_ (*p* >  0.1). For EDL muscles after the 200-cycle protocol, normalized forces were comparable to those after eccentric contraction protocol at 10% L_o_ for *mdx* (*p* >  0.2) and at 20% L_o_ for both WT (*p* >  0.8) and *mdx* (*p* >  0.8; Fig 6B). Normalized forces were higher than those at 30% L_o_ (*p* <  0.05 for WT and *mdx*). Only normalized forces of WT EDL after the 200-cycle were lower than those after the eccentric contraction at 10% (*p* <  0.05).

## Discussion

Two hypotheses regarding the *ex vivo* cycling protocol were examined in this study: 1) dystrophic soleus and EDL muscles from *mdx* mice would demonstrate damage when exposed to repeated changes in length, force and excitation that mimic those that corresponding human muscles experience in a cyclic human walking gait pattern; and 2) the damage to dystrophic soleus and EDL muscles revealed by the *ex vivo* gait cycling protocol would be less severe than a typical *ex vivo* eccentric contraction protocol [9]. Our results showed that only EDL muscles had significant losses in the isometric tetanic forces after the cycling protocols, while soleus muscles did not. However, both soleus and EDL muscles had significantly reduced peak forces in the last gait cycle (either 25^th^ or 200^th^) compared to the 1^st^ cycle during the gait cycling protocols. Our results also showed that, after the gait cycling protocols, soleus and EDL muscles produced higher or similar forces compared to those after the eccentric protocols, which suggested less severe or similar effects on contractile performance.

WT and *mdx* soleus and EDL muscles subjected to repeated gait cycles that mimicked the stresses, strains, and excitation patterns of these muscles during human walking gait revealed force losses in the EDL but not in the soleus. Although the dynamic peak forces during the gait cycling protocols were reduced in both soleus and EDL muscles (Fig 3), the isometric tetanic forces at 120Hz 1-min and 10-min post the cycling protocol, which reflect muscle force-generating capacity [9], were only reduced in EDL, but not in soleus (Fig 4). In fact, soleus muscles demonstrated increased tetanic force after the gait cycling protocols (see more discussion below). These outcomes suggested that it was likely that the decreased dynamic peak forces in EDL muscles resulted from a combination of fatigue and reduced force generating capacity, whereas fatigue may be the main cause for the decreases in the dynamic peak forces of soleus muscle during cycling protocols [41]. The different sensitivities of soleus and EDL to the cycling protocols are similar to those in eccentric contraction protocols, in which eccentric contractions at 10% and 20% L_o_ only induce minimal, comparable force losses in WT and *mdx* soleus muscles [42].The damage to the EDL but not to the soleus muscles after the cycling and eccentric contraction protocols may be due to more slow fiber, utrophin and cytoplasmic actin content in soleus compared to the EDL muscle [10,43]. In addition, while pennation angle is similar between the two muscles [44], more complex features of muscles’ architectures, such as fiber curvature and aponeurosis morphology may also play a role [45,46]. Our results together with previous studies [10,42] showing insensitivity of soleus to eccentric contraction protocols indicate that soleus muscle may not be a good candidate for pre-clinical studies that intend to understand disease progression and to evaluate the efficacy of treatments for DMD.

When comparing the gait cycling protocol to the eccentric contraction protocol [9], it appeared that the decrement in contractile performance, as indicated by force losses, is similar to that from the 10% and 20% L_o_ eccentric contraction protocols, but smaller than the 30% L_o_ eccentric contraction protocol (Fig 6). The eccentric contraction at 30% L_o_ had a large strain magnitude that can elicit substantial force losses both in soleus and EDL muscles [43]. Although soleus and EDL muscles in the gait cycling protocol had lengthening strains up to 30% L_o_, they were under submaximal activation during the majority of a gait cycle [15]. As activation level strongly affects the magnitude of force losses after eccentric contractions, in the gait cycling protocols the submaximal activation yielded less than maximum isometric forces, which potentially resulted in smaller force losses or contractile impairment [47,48]. Other than the soleus muscles in the 25-cycle protocol that showed some increased forces, the force losses in soleus from the cycling protocol were close to the negligeable losses elicited from eccentric contractions at 10% L_o_ in soleus. For EDL muscles, the force loss from the cycling protocol was close to that elicited from eccentric contractions at 20% L_o_, which may suggest that the eccentric contractions at 20% L_o_ with a strain rate of 50% L_o_/s and 5 repetitions may reflect the effects of human walking gait experienced by EDL muscles. Surprisingly, our results showed that eccentric contractions at 30% L_o_ induced more force losses in WT than in *mdx* soleus muscles (Fig 2). There were only a limited number of studies that examined the force losses of soleus from *mdx* mice after eccentric contractions, which typically lengthened muscle up to 20% L_o_ and showed no differences between the WT and *mdx* [10,42,49,50], consistent to our results at 10% and 20% L_o_. The greater force losses in WT than in *mdx* soleus muscles at 30% L_o_ may be due to the a greater peak active force produced by the WT soleus during eccentric contractions at this magnitude, which led to the disruptions of sarcomere and/or the excitation-contraction coupling process that caused the greater force loss [51,52].

There were no differences between the genotypes in the responses of soleus and EDL muscles to the gait cycling protocols (Fig 3), which may be due to the differences in histopathology between hindlimb muscles in *mdx* mice and lower limb muscles in patients with DMD. Given that the hindlimb muscles in *mdx* mice are much less affected than human DMD lower limb muscles [53], it is plausible that when physiologically relevant loadings that mimicked human walking were applied, no significant differences were shown between WT and *mdx* mice. Mimicking stresses and strains in dystrophic muscles from mouse models that better recapitulate the histopathology of DMD, such as the D2.*mdx* [54], may elicit divergent responses from WT and dystrophic muscles. In addition, the cycling protocol can be slightly modified to mimic the walking pattern of boys with DMD, which have demonstrated some kinematic and kinetic differences from healthy boys and adult males [55–58]. Or, the cycling protocol can mimic daily activities that demand more stressful eccentric contractions from lower limb muscles, such as walking downhill or descending stairs, than level ground walking [59,60], to elicit different responses from WT and *mdx* muscles. Although traditional eccentric contraction protocols elicit more force losses in fast-twitch muscles between WT and *mdx* mice [37], the more physiologically relevant cycling protocol may improve the transferability of preclinical findings in mouse models of DMD to patients.

The increased tetanic force in the soleus muscle after the gait cycling protocol was unexpected. However, there are several possible mechanisms that may have led to this observation (Fig 4). One possible mechanism may be the residual force enhancement potentially induced by the elasticity of titin [61]. Residual force enhancement contributes to increased isometric force after cycles of shortening and lengthening [62,63] and the increased isometric force can be greater than the maximum isometric force at L_o_.[64]. So, it is plausible that after repeated shortening and lengthening in the gait cycling protocol, soleus muscles in our study showed increased isometric force over the force at L_o_ before the protocol due to residual force enhancement. However, it has been reported that the enhanced force disappears if electrical stimulation to the muscle is interrupted [65]. Because in our experiment the soleus force was measured after a 1-minute rest following the cycling protocol, residual force enhancement may not be responsible. Another possible mechanism is force potentiation related to phosphorylation of the myosin regulatory light chains due to prior activation that increased the Ca^2 +^ sensitivity of contraction [66]. However, force potentiation has been mostly observed in muscles dominated with predominant fast-twitch fibers, rather than those with mostly slow-twitch fibers, like soleus [67]. In addition, the fast-twitch EDL muscle did not show signs of force potentiation after the gait cycling protocol. Thus, it seems unlikely that the force potentiation increased soleus force observed after the gait cycling protocols.

It was surprising that, even though gait cycling protocols lead to loss in force production, we did not observe significant differences in procion orange dye uptake (Fig 5). Our results indicated no difference between non-injured and injured muscles across genotype. While previous work has reported differences in sarcolemmal leakiness measured via procion orange dye uptake in these limb muscles between *mdx* and WT mice after eccentric contractions, our results from muscles subjected to the gait cycling indicated *mdx* samples generally had a large dye uptake that was higher in non-injured samples [8,68]. Future work is necessary to investigate alternative markers of sarcolemmal damage such as Evan’s Blue Dye or H&E staining [69].

Current skeletal muscle immunohistochemistry segmentation methods are limited and may be highly variable due to a variety of factors. Detailed skeletal muscle immunohistochemistry segmentation is often manual, observer dependent, and time-consuming [70]. This analysis could vary based on the observer’s visual acuity, as well as dexterity to quantify the fiber morphology such as cross-sectional area or shape through manual segmentation [71,72]. Additionally, manual analysis can introduce user bias when doing a comparative study as the observer may not be blinded to the condition. The current literature does not provide detailed methods of quantification such as what intensity level or the amount of fiber cross-section positively stained is determined as a positive fiber, allowing for variability in inter-observer counts and non-standard implementation [9,73,74]. Recent advances in image segmentation techniques have resulted in several opensource platforms; however, these platforms still require a large degree of oversight which is not easily viable in large experimental studies [75,76]. Due to these limitations, we designed and employed a segmentation algorithm to standardize the quantification of dye across samples. Our algorithm was compared to the gold standard manual quantification from two skilled analysts for randomly selected samples with great agreement (within 3%).

The gait cycling protocol does have some limitations. First, when mimicking walking gait, to ensure that the forces in mouse soleus and EDL muscles were comparable to the corresponding human muscles, voltage modulation was needed for each individual muscle. With some practice, this voltage modulation could be completed within several gait cycles [17]. Although this extra step may have increased the time duration of the cycling protocol, the viability of the muscles was maintained, because the isometric tetanic forces did not differ within, 1-min after, or 10-min after the 200-cycle protocol (Fig 4). Second, fatigue may contribute to the decrease of dynamic peak forces in the cycling protocol. Thus, isometric tetani and appropriate rests after the gait mimicking cycles are needed to probe the actual force loss due to the damage to the muscle contractile properties by the protocol.

This study explored the use of the gait cycling protocol to mimic the stresses, strains and excitation patterns of human soleus and EDL muscles during level ground walking in the corresponding muscles in WT and *mdx* mice. By closely replicating *in vivo* human muscle functions in activities, this protocol reduces the biomechanical differences in muscle function between humans and mice that may hinder the application of mouse models in pre-clinical studies [6]. The damaging effects to the contractile performance from the cycling protocol were similar to those from the eccentric contractions at 20% L_o_. This outcome suggests potential of the cycling protocol as a valuable experimental approach to better understand disease progression and to screen and evaluate efficacy of novel therapeutics for DMD. Future studies can further apply cycling protocols to replicate muscle function in activities involving more strenuous eccentric contractions, such as walking down stairs or downhill [59], to better understand the impact on dystrophic muscles. Our results also indicated the need for future studies to use mouse models that more closely reflect the pathological features of DMD [77]. The cycling protocol with the ability to mimic the time-varying strain and activation patterns of human muscles in locomotion in smaller mouse muscles may also have extended use for understanding muscle mechanics in general, since it more closely replicates *in vivo* muscle function in locomotion than the traditional work loop technique [78]. Indeed, this approach has been used to demonstrate the importance of muscle force-velocity properties, activation intensity and spindle abundance on the appropriate interpretations of *in vivo* muscle functions [79–81]. Given that details of the gait cycling protocol were described in our Methods paper [17] and modified stimulator (Model 701C) is available through Aurora Scientific Inc., we expect that more studies using this or a similar approach will come out in the near future.

## Supporting information

S1 Fig The typical force responses during the eccentric contraction protocol for WT EDL muscle at 10% (A), 20% (B) and 30% (C) L_o_, and for mdx EDL muscle at 10% (D), 20% (E) and 30% (F) L_o_. The length changes used in the eccentric contractions at 10%, 20% and 30% Lo are shown in (G), (H) and (I), respectively. For clarity only the responses of the 1^st^, 3^rd^ and 5^th^ eccentric contractions are shown.
(DOCX)
