# Supplementary material for: A novel ex vivo protocol that mimics length and excitation changes of human muscles during walking induces force losses in EDL but not in soleus of mdx mice
Source: PLoS One. 2025 Apr 7;20(4):e0320901. doi: 10.1371/journal.pone.0320901 (PMC11975108; doi:10.1371/journal.pone.0320901)
Supplement: S1 Fig — (A), 20% (B) and 30% (C) Lo, and for mdx EDL muscle at 10% (D), 20% (E) and 30% (F) Lo. The length changes used in the eccentric contractions at 10%, 20% and 30% Lo are shown in (G), (H) and (I), respectively. For clarity only the responses of the 1st, 3rd and 5th eccentric contractions are shown. (DOCX) [file pone.0320901.s001.docx]

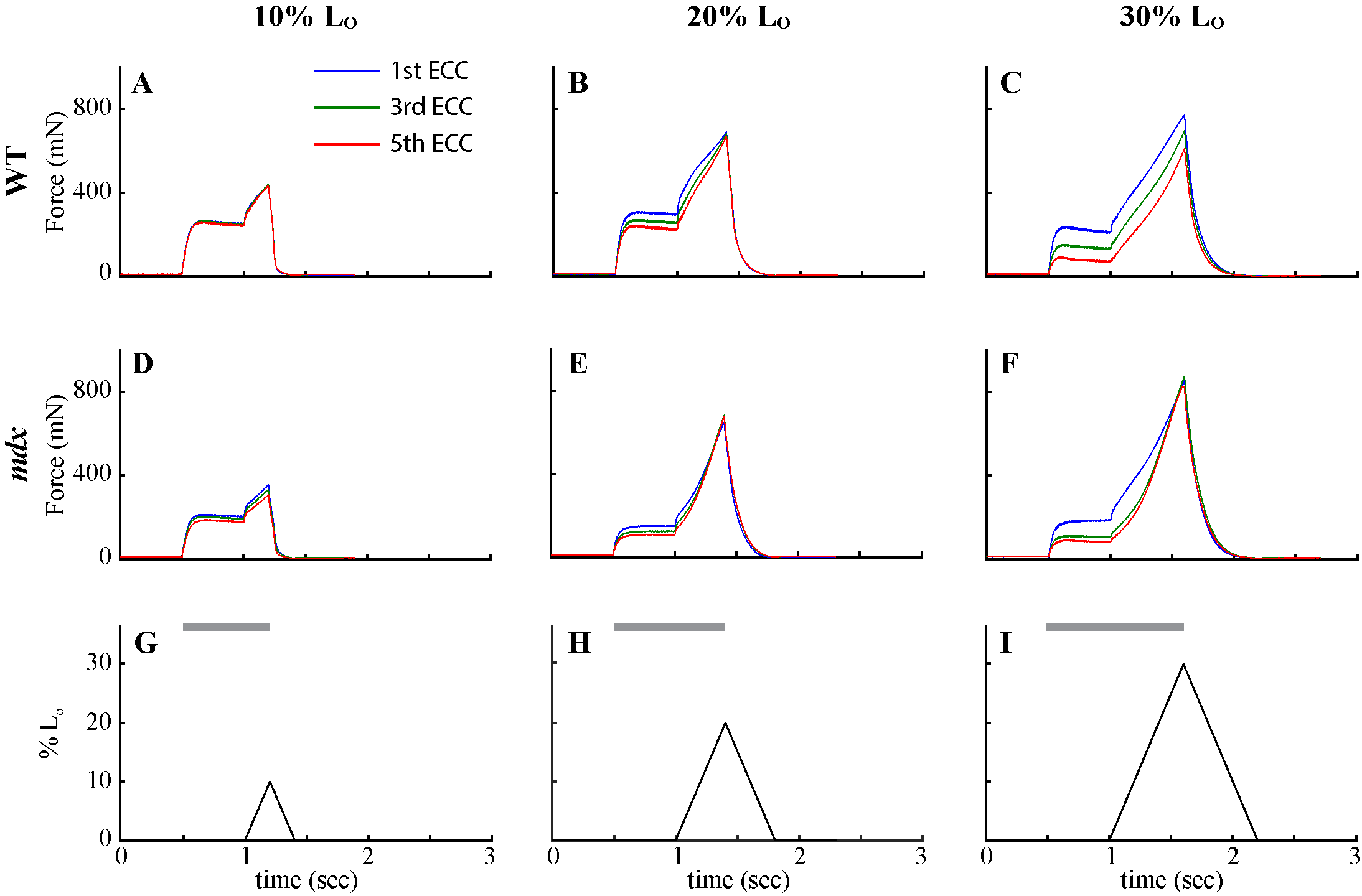


S1 Fig. The typical force responses during the eccentric contraction protocol for WT EDL muscle at 10% (A), 20% (B) and 30% (C) L_o_, and for *mdx* EDL muscle at 10% (D), 20% (E) and 30% (F) L_o_. The length changes used in the eccentric contractions at 10%, 20% and 30% L_o_ are shown in (G), (H) and (I), respectively. For clarity only the responses of the 1^st^, 3^rd^ and 5^th^ eccentric contractions are shown.
